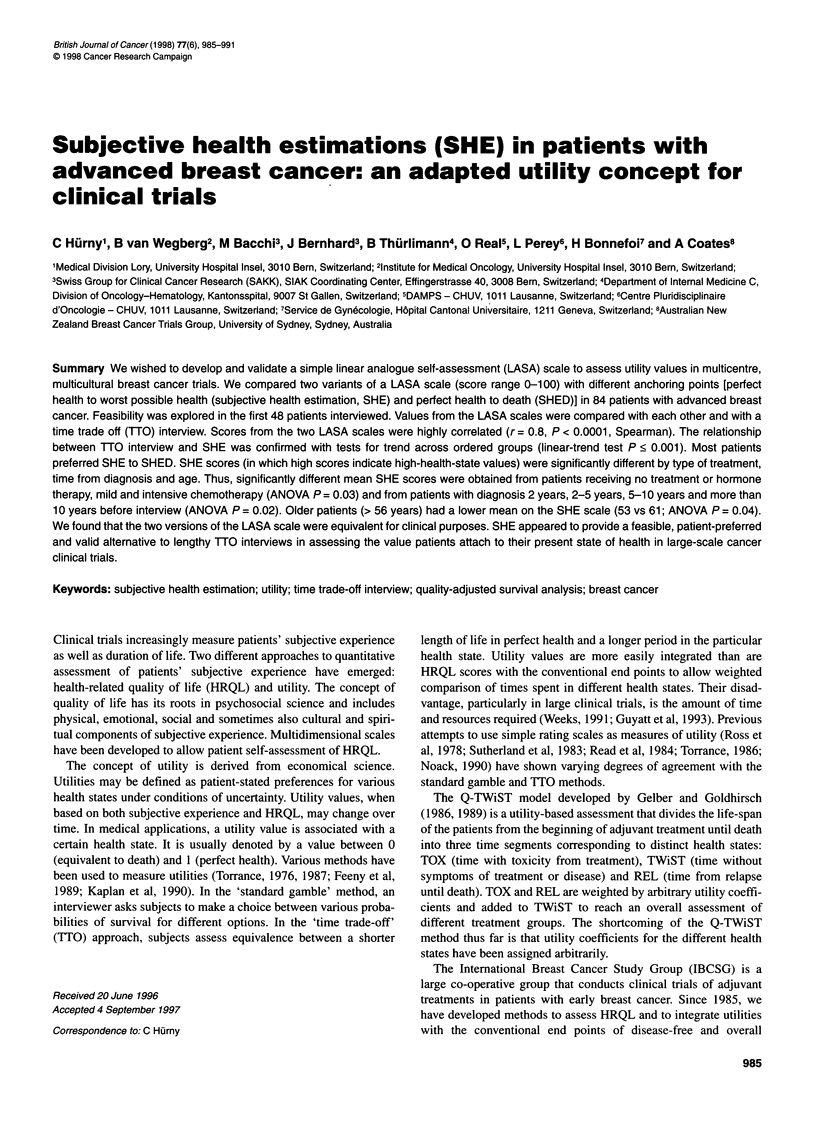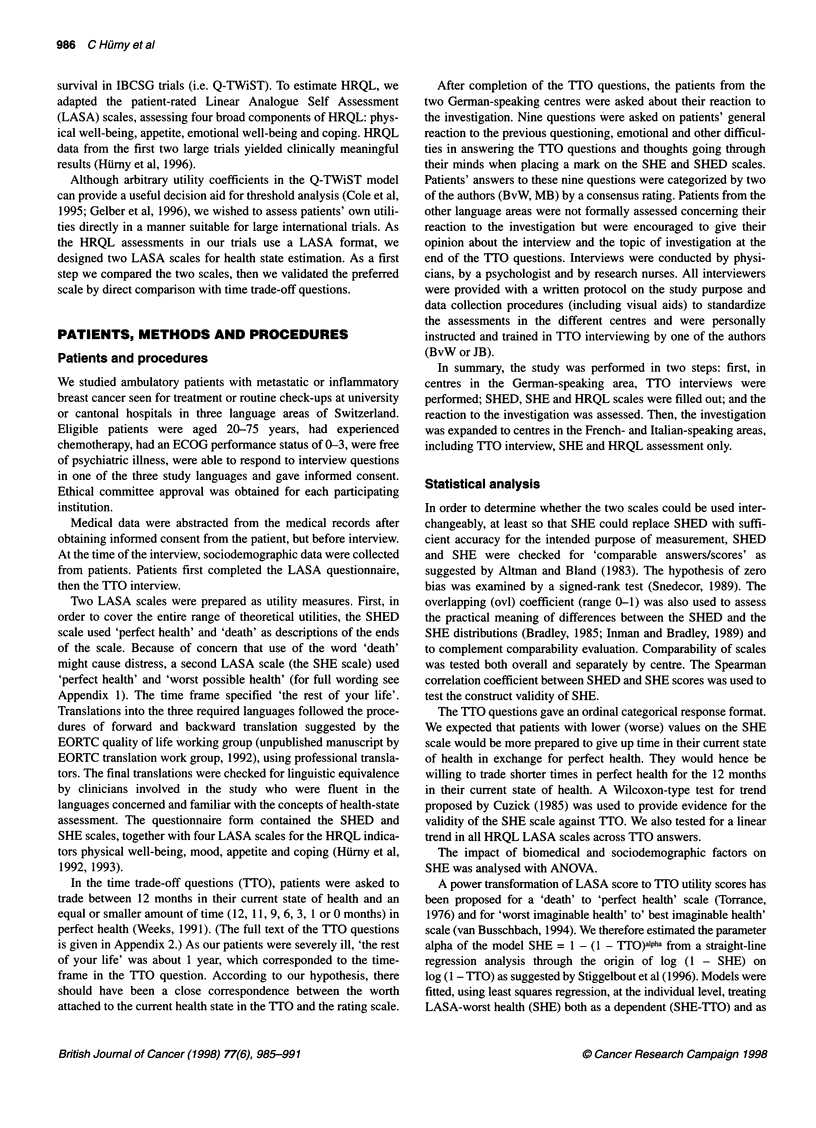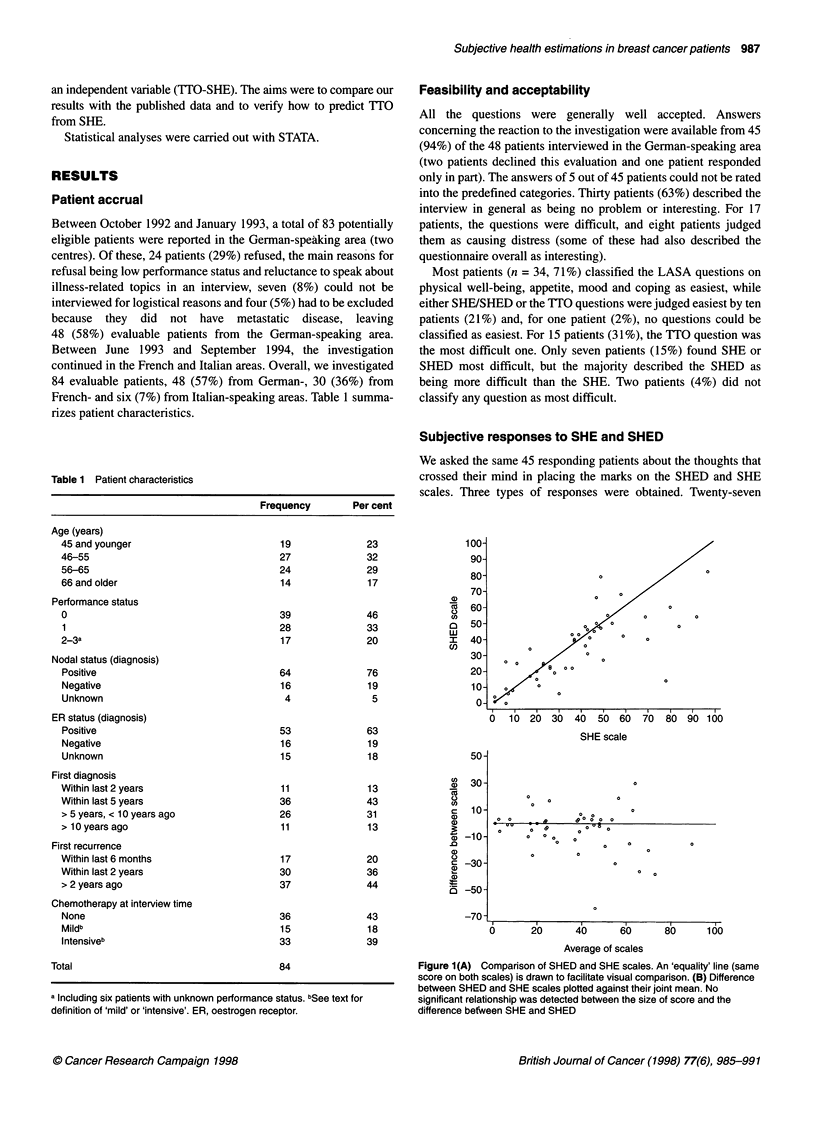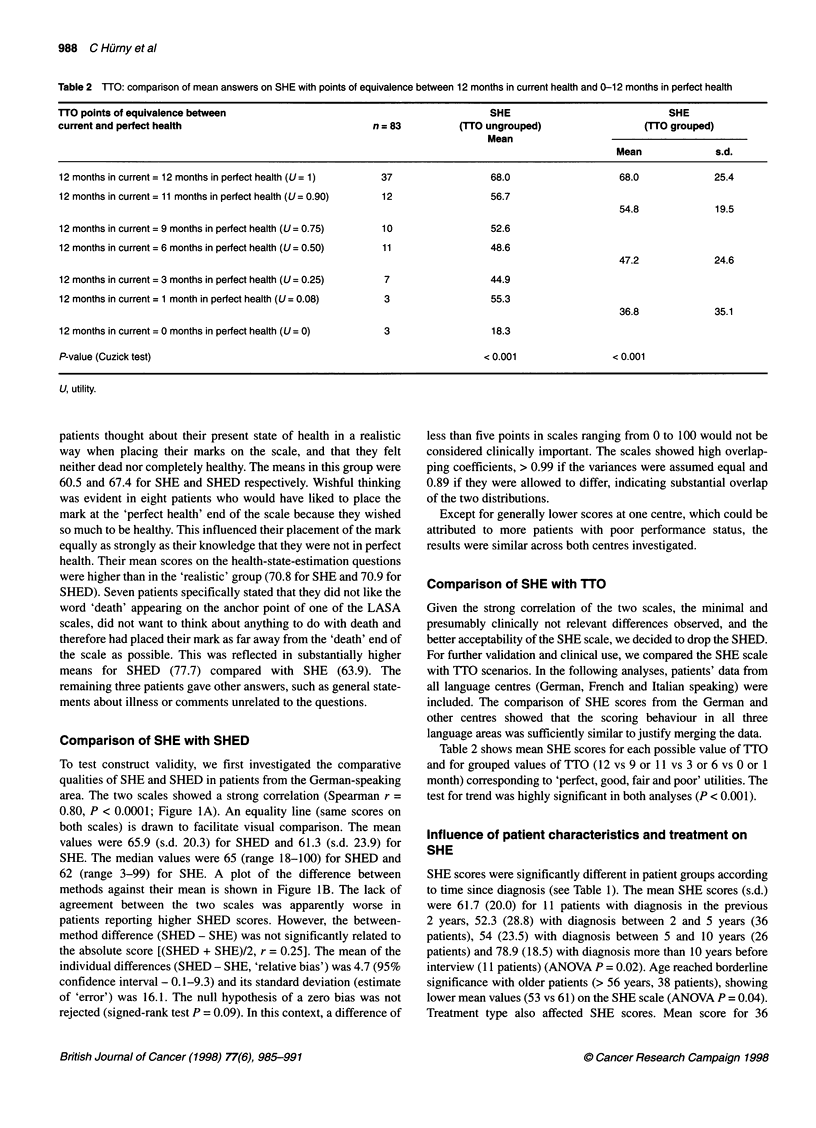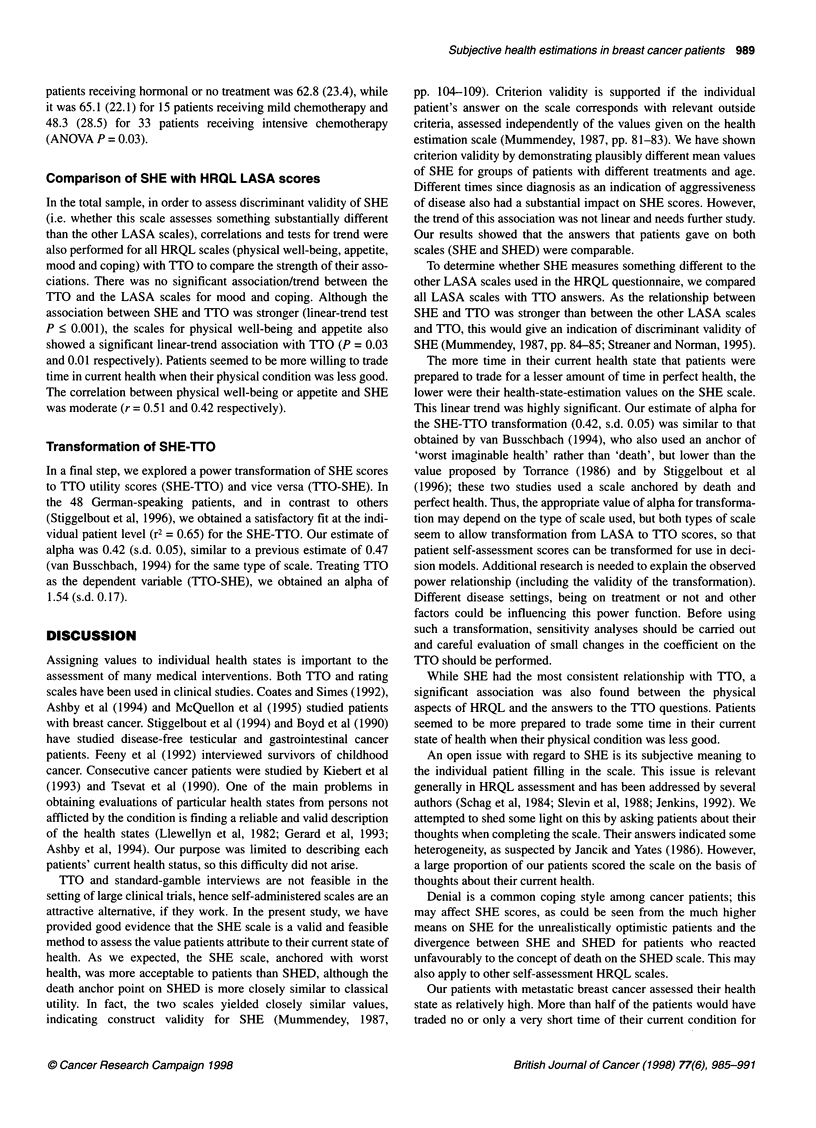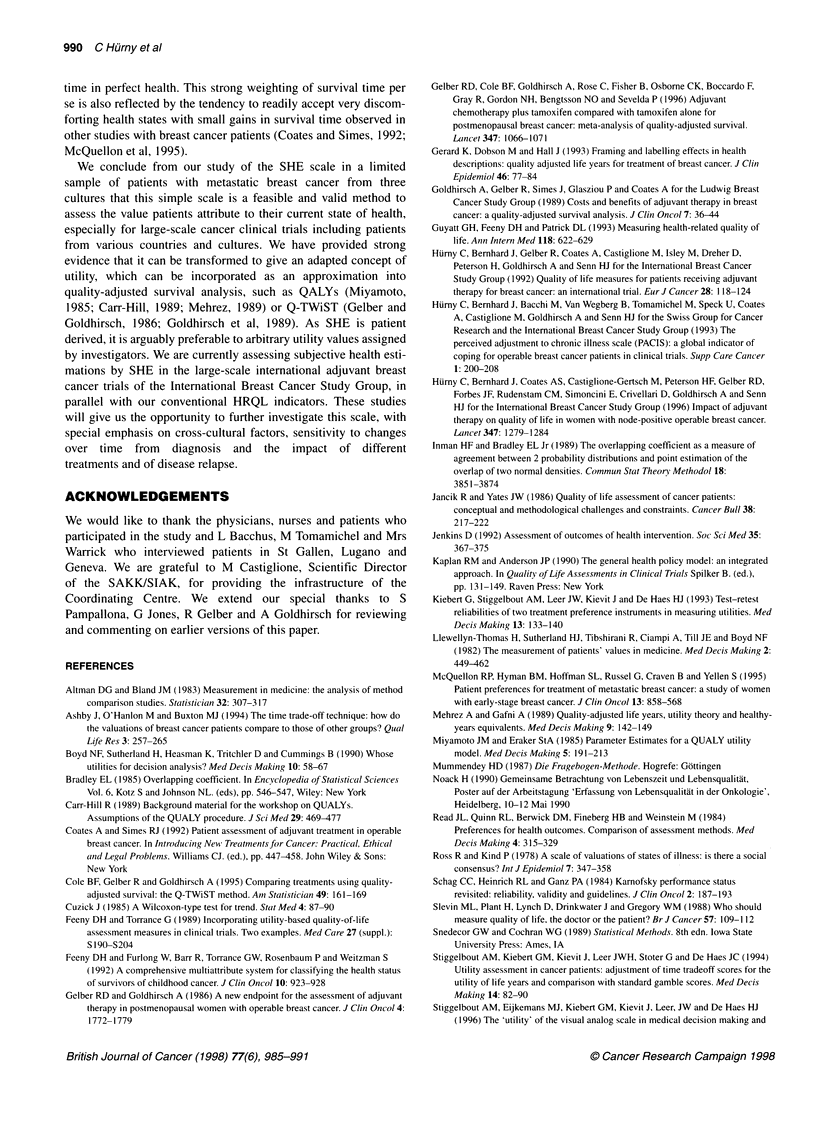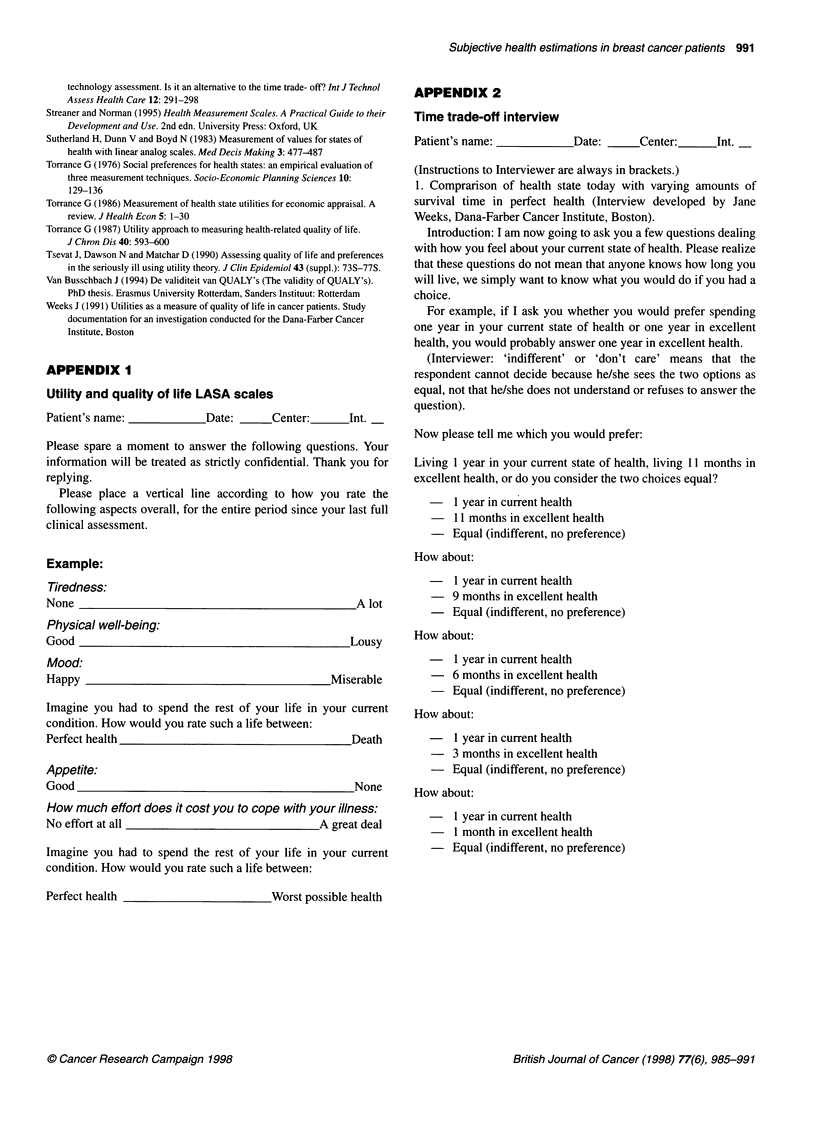# Subjective health estimations (SHE) in patients with advanced breast cancer: an adapted utility concept for clinical trials.

**DOI:** 10.1038/bjc.1998.162

**Published:** 1998-03

**Authors:** C. HÃ¼rny, B. van Wegberg, M. Bacchi, J. Bernhard, B. ThÃ¼rlimann, O. Real, L. Perey, H. Bonnefoi, A. Coates

**Affiliations:** Medical Division Lory, University Hospital Insel, Bern, Switzerland.

## Abstract

We wished to develop and validate a simple linear analogue self-assessment (LASA) scale to assess utility values in multicentre, multicultural breast cancer trials. We compared two variants of a LASA scale (score range 0-100) with different anchoring points [perfect health to worst possible health (subjective health estimation, SHE) and perfect health to death (SHED)] in 84 patients with advanced breast cancer. Feasibility was explored in the first 48 patients interviewed. Values from the LASA scales were compared with each other and with a time trade off (TTO) interview. Scores from the two LASA scales were highly correlated (r=0.8, P < 0.0001, Spearman). The relationship between TTO interview and SHE was confirmed with tests for trend across ordered groups (linear-trend test P < 0.001). Most patients preferred SHE to SHED. SHE scores (in which high scores indicate high-health-state values) were significantly different by type of treatment, time from diagnosis and age. Thus, significantly different mean SHE scores were obtained from patients receiving no treatment or hormone therapy, mild and intensive chemotherapy (ANOVA P=0.03) and from patients with diagnosis 2 years, 2-5 years, 5-10 years and more than 10 years before interview (ANOVA P=0.02). Older patients (> 56 years) had a lower mean on the SHE scale (53 vs 61; ANOVA P=0.04). We found that the two versions of the LASA scale were equivalent for clinical purposes. SHE appeared to provide a feasible, patient-preferred and valid alternative to lengthy TTO interviews in assessing the value patients attach to their present state of health in large-scale cancer clinical trials.


British Journal of Cancer (1998) 77(6), 985–991
© 1998 Cancer Research Campaign

### Subjective health estimations (SHE) in patients with advanced breast cancer: an adapted utility concept for clinical trials

**C Hürny[1], B van Wegberg[2], M Bacchi[3], J Bernhard[3], B Thürlimann[4], O Real[5], L Perey[6], H Bonnefoi[7] and A Coates[8]**

[1]Medical Division Lory, University Hospital Insel, 3010 Bern, Switzerland; [2]Institute for Medical Oncology, University Hospital Insel, 3010 Bern, Switzerland; [3]Swiss Group for Clinical Cancer Research (SAKK), SIAK Coordinating Center, Effingerstrasse 40, 3008 Bern, Switzerland; [4]Department of Internal Medicine C, Division of Oncology–Hematology, Kantonsspital, 9007 St Gallen, Switzerland; [5]DAMPS – CHUV, 1011 Lausanne, Switzerland; [6]Centre Pluridisciplinaire d'Oncologie – CHUV, 1011 Lausanne, Switzerland; [7]Service de Gynécologie, Hôpital Cantonal Universitaire, 1211 Geneva, Switzerland; [8]Australian New Zealand Breast Cancer Trials Group, University of Sydney, Sydney, Australia

**Summary** We wished to develop and validate a simple linear analogue self-assessment (LASA) scale to assess utility values in multicentre, multicultural breast cancer trials. We compared two variants of a LASA scale (score range 0–100) with different anchoring points [perfect health to worst possible health (subjective health estimation, SHE) and perfect health to death (SHED)] in 84 patients with advanced breast cancer. Feasibility was explored in the first 48 patients interviewed. Values from the LASA scales were compared with each other and with a time trade off (TTO) interview. Scores from the two LASA scales were highly correlated ($r = 0.8$, $P < 0.0001$, Spearman). The relationship between TTO interview and SHE was confirmed with tests for trend across ordered groups (linear-trend test $P \leq 0.001$). Most patients preferred SHE to SHED. SHE scores (in which high scores indicate high-health-state values) were significantly different by type of treatment, time from diagnosis and age. Thus, significantly different mean SHE scores were obtained from patients receiving no treatment or hormone therapy, mild and intensive chemotherapy (ANOVA $P = 0.03$) and from patients with diagnosis 2 years, 2–5 years, 5–10 years and more than 10 years before interview (ANOVA $P = 0.02$). Older patients (> 56 years) had a lower mean on the SHE scale (53 vs 61; ANOVA $P = 0.04$). We found that the two versions of the LASA scale were equivalent for clinical purposes. SHE appeared to provide a feasible, patient-preferred and valid alternative to lengthy TTO interviews in assessing the value patients attach to their present state of health in large-scale cancer clinical trials.

**Keywords:** subjective health estimation; utility; time trade-off interview; quality-adjusted survival analysis; breast cancer

Clinical trials increasingly measure patients' subjective experience as well as duration of life. Two different approaches to quantitative assessment of patients' subjective experience have emerged: health-related quality of life (HRQL) and utility. The concept of quality of life has its roots in psychosocial science and includes physical, emotional, social and sometimes also cultural and spiritual components of subjective experience. Multidimensional scales have been developed to allow patient self-assessment of HRQL.

The concept of utility is derived from economical science. Utilities may be defined as patient-stated preferences for various health states under conditions of uncertainty. Utility values, when based on both subjective experience and HRQL, may change over time. In medical applications, a utility value is associated with a certain health state. It is usually denoted by a value between 0 (equivalent to death) and 1 (perfect health). Various methods have been used to measure utilities (Torrance, 1976, 1987; Feeny et al, 1989; Kaplan et al, 1990). In the 'standard gamble' method, an interviewer asks subjects to make a choice between various probabilities of survival for different options. In the 'time trade-off' (TTO) approach, subjects assess equivalence between a shorter length of life in perfect health and a longer period in the particular health state. Utility values are more easily integrated than are HRQL scores with the conventional end points to allow weighted comparison of times spent in different health states. Their disadvantage, particularly in large clinical trials, is the amount of time and resources required (Weeks, 1991; Guyatt et al, 1993). Previous attempts to use simple rating scales as measures of utility (Ross et al, 1978; Sutherland et al, 1983; Read et al, 1984; Torrance, 1986; Noack, 1990) have shown varying degrees of agreement with the standard gamble and TTO methods.

The Q-TWiST model developed by Gelber and Goldhirsch (1986, 1989) is a utility-based assessment that divides the life-span of the patients from the beginning of adjuvant treatment until death into three time segments corresponding to distinct health states: TOX (time with toxicity from treatment), TWiST (time without symptoms of treatment or disease) and REL (time from relapse until death). TOX and REL are weighted by arbitrary utility coefficients and added to TWiST to reach an overall assessment of different treatment groups. The shortcoming of the Q-TWiST method thus far is that utility coefficients for the different health states have been assigned arbitrarily.

The International Breast Cancer Study Group (IBCSG) is a large co-operative group that conducts clinical trials of adjuvant treatments in patients with early breast cancer. Since 1985, we have developed methods to assess HRQL and to integrate utilities with the conventional end points of disease-free and overall

*Received 20 June 1996*
*Accepted 4 September 1997*

*Correspondence to:* C Hürny survival in IBCSG trials (i.e. Q-TWiST). To estimate HRQL, we adapted the patient-rated Linear Analogue Self Assessment (LASA) scales, assessing four broad components of HRQL: physical well-being, appetite, emotional well-being and coping. HRQL data from the first two large trials yielded clinically meaningful results (Hürny et al, 1996).

Although arbitrary utility coefficients in the Q-TWiST model can provide a useful decision aid for threshold analysis (Cole et al, 1995; Gelber et al, 1996), we wished to assess patients' own utilities directly in a manner suitable for large international trials. As the HRQL assessments in our trials use a LASA format, we designed two LASA scales for health state estimation. As a first step we compared the two scales, then we validated the preferred scale by direct comparison with time trade-off questions.

### PATIENTS, METHODS AND PROCEDURES

### Patients and procedures

We studied ambulatory patients with metastatic or inflammatory breast cancer seen for treatment or routine check-ups at university or cantonal hospitals in three language areas of Switzerland. Eligible patients were aged 20–75 years, had experienced chemotherapy, had an ECOG performance status of 0–3, were free of psychiatric illness, were able to respond to interview questions in one of the three study languages and gave informed consent. Ethical committee approval was obtained for each participating institution.

Medical data were abstracted from the medical records after obtaining informed consent from the patient, but before interview. At the time of the interview, sociodemographic data were collected from patients. Patients first completed the LASA questionnaire, then the TTO interview.

Two LASA scales were prepared as utility measures. First, in order to cover the entire range of theoretical utilities, the SHED scale used 'perfect health' and 'death' as descriptions of the ends of the scale. Because of concern that use of the word 'death' might cause distress, a second LASA scale (the SHE scale) used 'perfect health' and 'worst possible health' (for full wording see Appendix 1). The time frame specified 'the rest of your life'. Translations into the three required languages followed the procedures of forward and backward translation suggested by the EORTC quality of life working group (unpublished manuscript by EORTC translation work group, 1992), using professional translators. The final translations were checked for linguistic equivalence by clinicians involved in the study who were fluent in the languages concerned and familiar with the concepts of health-state assessment. The questionnaire form contained the SHED and SHE scales, together with four LASA scales for the HRQL indicators physical well-being, mood, appetite and coping (Hürny et al, 1992, 1993).

In the time trade-off questions (TTO), patients were asked to trade between 12 months in their current state of health and an equal or smaller amount of time (12, 11, 9, 6, 3, 1 or 0 months) in perfect health (Weeks, 1991). (The full text of the TTO questions is given in Appendix 2.) As our patients were severely ill, 'the rest of your life' was about 1 year, which corresponded to the timeframe in the TTO question. According to our hypothesis, there should have been a close correspondence between the worth attached to the current health state in the TTO and the rating scale.

After completion of the TTO questions, the patients from the two German-speaking centres were asked about their reaction to the investigation. Nine questions were asked on patients' general reaction to the previous questioning, emotional and other difficulties in answering the TTO questions and thoughts going through their minds when placing a mark on the SHE and SHED scales. Patients' answers to these nine questions were categorized by two of the authors (BvW, MB) by a consensus rating. Patients from the other language areas were not formally assessed concerning their reaction to the investigation but were encouraged to give their opinion about the interview and the topic of investigation at the end of the TTO questions. Interviews were conducted by physicians, by a psychologist and by research nurses. All interviewers were provided with a written protocol on the study purpose and data collection procedures (including visual aids) to standardize the assessments in the different centres and were personally instructed and trained in TTO interviewing by one of the authors (BvW or JB).

In summary, the study was performed in two steps: first, in centres in the German-speaking area, TTO interviews were performed; SHED, SHE and HRQL scales were filled out; and the reaction to the investigation was assessed. Then, the investigation was expanded to centres in the French- and Italian-speaking areas, including TTO interview, SHE and HRQL assessment only.

### Statistical analysis

In order to determine whether the two scales could be used interchangeably, at least so that SHE could replace SHED with sufficient accuracy for the intended purpose of measurement, SHED and SHE were checked for 'comparable answers/scores' as suggested by Altman and Bland (1983). The hypothesis of zero bias was examined by a signed-rank test (Snedecor, 1989). The overlapping (ovl) coefficient (range 0–1) was also used to assess the practical meaning of differences between the SHED and the SHE distributions (Bradley, 1985; Inman and Bradley, 1989) and to complement comparability evaluation. Comparability of scales was tested both overall and separately by centre. The Spearman correlation coefficient between SHED and SHE scores was used to test the construct validity of SHE.

The TTO questions gave an ordinal categorical response format. We expected that patients with lower (worse) values on the SHE scale would be more prepared to give up time in their current state of health in exchange for perfect health. They would hence be willing to trade shorter times in perfect health for the 12 months in their current state of health. A Wilcoxon-type test for trend proposed by Cuzick (1985) was used to provide evidence for the validity of the SHE scale against TTO. We also tested for a linear trend in all HRQL LASA scales across TTO answers.

The impact of biomedical and sociodemographic factors on SHE was analysed with ANOVA.

A power transformation of LASA score to TTO utility scores has been proposed for a 'death' to 'perfect health' scale (Torrance, 1976) and for 'worst imaginable health' to' best imaginable health' scale (van Busschbach, 1994). We therefore estimated the parameter alpha of the model $\text{SHE} = 1 - (1 - \text{TTO})^{\text{alpha}}$ from a straight-line regression analysis through the origin of log (1 – SHE) on log (1 – TTO) as suggested by Stiggelbout et al (1996). Models were fitted, using least squares regression, at the individual level, treating LASA-worst health (SHE) both as a dependent (SHE-TTO) and as

 © Cancer Research Campaign 1998

an independent variable (TTO-SHE). The aims were to compare our results with the published data and to verify how to predict TTO from SHE.

Statistical analyses were carried out with STATA.

### RESULTS

### Patient accrual

Between October 1992 and January 1993, a total of 83 potentially eligible patients were reported in the German-speaking area (two centres). Of these, 24 patients (29%) refused, the main reasons for refusal being low performance status and reluctance to speak about illness-related topics in an interview, seven (8%) could not be interviewed for logistical reasons and four (5%) had to be excluded because they did not have metastatic disease, leaving 48 (58%) evaluable patients from the German-speaking area. Between June 1993 and September 1994, the investigation continued in the French and Italian areas. Overall, we investigated 84 evaluable patients, 48 (57%) from German-, 30 (36%) from French- and six (7%) from Italian-speaking areas. Table 1 summarizes patient characteristics.

**Table 1** Patient characteristics

| | Frequency | Per cent |
|---|---|---|
| Age (years) | | |
| 45 and younger | 19 | 23 |
| 46–55 | 27 | 32 |
| 56–65 | 24 | 29 |
| 66 and older | 14 | 17 |
| Performance status | | |
| 0 | 39 | 46 |
| 1 | 28 | 33 |
| 2–3[a] | 17 | 20 |
| Nodal status (diagnosis) | | |
| Positive | 64 | 76 |
| Negative | 16 | 19 |
| Unknown | 4 | 5 |
| ER status (diagnosis) | | |
| Positive | 53 | 63 |
| Negative | 16 | 19 |
| Unknown | 15 | 18 |
| First diagnosis | | |
| Within last 2 years | 11 | 13 |
| Within last 5 years | 36 | 43 |
| > 5 years, < 10 years ago | 26 | 31 |
| > 10 years ago | 11 | 13 |
| First recurrence | | |
| Within last 6 months | 17 | 20 |
| Within last 2 years | 30 | 36 |
| > 2 years ago | 37 | 44 |
| Chemotherapy at interview time | | |
| None | 36 | 43 |
| Mild[b] | 15 | 18 |
| Intensive[b] | 33 | 39 |
| Total | 84 | |

[a] Including six patients with unknown performance status. [b]See text for definition of 'mild' or 'intensive'. ER, oestrogen receptor.

### Feasibility and acceptability

All the questions were generally well accepted. Answers concerning the reaction to the investigation were available from 45 (94%) of the 48 patients interviewed in the German-speaking area (two patients declined this evaluation and one patient responded only in part). The answers of 5 out of 45 patients could not be rated into the predefined categories. Thirty patients (63%) described the interview in general as being no problem or interesting. For 17 patients, the questions were difficult, and eight patients judged them as causing distress (some of these had also described the questionnaire overall as interesting).

Most patients ($n$ = 34, 71%) classified the LASA questions on physical well-being, appetite, mood and coping as easiest, while either SHE/SHED or the TTO questions were judged easiest by ten patients (21%) and, for one patient (2%), no questions could be classified as easiest. For 15 patients (31%), the TTO question was the most difficult one. Only seven patients (15%) found SHE or SHED most difficult, but the majority described the SHED as being more difficult than the SHE. Two patients (4%) did not classify any question as most difficult.

### Subjective responses to SHE and SHED

We asked the same 45 responding patients about the thoughts that crossed their mind in placing the marks on the SHED and SHE scales. Three types of responses were obtained. Twenty-seven

**Figure 1(A)** Comparison of SHED and SHE scales. An 'equality' line (same score on both scales) is drawn to facilitate visual comparison. **(B)** Difference between SHED and SHE scales plotted against their joint mean. No significant relationship was detected between the size of score and the difference between SHE and SHED

**Table 2** TTO: comparison of mean answers on SHE with points of equivalence between 12 months in current health and 0–12 months in perfect health

| TTO points of equivalence between current and perfect health | n = 83 | SHE (TTO ungrouped) Mean | SHE (TTO grouped) Mean | s.d. |
|---|---|---|---|---|
| 12 months in current = 12 months in perfect health (*U* = 1) | 37 | 68.0 | 68.0 | 25.4 |
| 12 months in current = 11 months in perfect health (*U* = 0.90) | 12 | 56.7 | 54.8 | 19.5 |
| 12 months in current = 9 months in perfect health (*U* = 0.75) | 10 | 52.6 | | |
| 12 months in current = 6 months in perfect health (*U* = 0.50) | 11 | 48.6 | 47.2 | 24.6 |
| 12 months in current = 3 months in perfect health (*U* = 0.25) | 7 | 44.9 | | |
| 12 months in current = 1 month in perfect health (*U* = 0.08) | 3 | 55.3 | 36.8 | 35.1 |
| 12 months in current = 0 months in perfect health (*U* = 0) | 3 | 18.3 | | |
| *P*-value (Cuzick test) | | < 0.001 | < 0.001 | |

*U*, utility.

patients thought about their present state of health in a realistic way when placing their marks on the scale, and that they felt neither dead nor completely healthy. The means in this group were 60.5 and 67.4 for SHE and SHED respectively. Wishful thinking was evident in eight patients who would have liked to place the mark at the 'perfect health' end of the scale because they wished so much to be healthy. This influenced their placement of the mark equally as strongly as their knowledge that they were not in perfect health. Their mean scores on the health-state-estimation questions were higher than in the 'realistic' group (70.8 for SHE and 70.9 for SHED). Seven patients specifically stated that they did not like the word 'death' appearing on the anchor point of one of the LASA scales, did not want to think about anything to do with death and therefore had placed their mark as far away from the 'death' end of the scale as possible. This was reflected in substantially higher means for SHED (77.7) compared with SHE (63.9). The remaining three patients gave other answers, such as general statements about illness or comments unrelated to the questions.

### Comparison of SHE with SHED

To test construct validity, we first investigated the comparative qualities of SHE and SHED in patients from the German-speaking area. The two scales showed a strong correlation (Spearman $r$ = 0.80, $P < 0.0001$; Figure 1A). An equality line (same scores on both scales) is drawn to facilitate visual comparison. The mean values were 65.9 (s.d. 20.3) for SHED and 61.3 (s.d. 23.9) for SHE. The median values were 65 (range 18–100) for SHED and 62 (range 3–99) for SHE. A plot of the difference between methods against their mean is shown in Figure 1B. The lack of agreement between the two scales was apparently worse in patients reporting higher SHED scores. However, the between-method difference (SHED – SHE) was not significantly related to the absolute score [(SHED + SHE)/2, $r = 0.25$]. The mean of the individual differences (SHED – SHE, 'relative bias') was 4.7 (95% confidence interval – 0.1–9.3) and its standard deviation (estimate of 'error') was 16.1. The null hypothesis of a zero bias was not rejected (signed-rank test $P = 0.09$). In this context, a difference of less than five points in scales ranging from 0 to 100 would not be considered clinically important. The scales showed high overlapping coefficients, > 0.99 if the variances were assumed equal and 0.89 if they were allowed to differ, indicating substantial overlap of the two distributions.

Except for generally lower scores at one centre, which could be attributed to more patients with poor performance status, the results were similar across both centres investigated.

### Comparison of SHE with TTO

Given the strong correlation of the two scales, the minimal and presumably clinically not relevant differences observed, and the better acceptability of the SHE scale, we decided to drop the SHED. For further validation and clinical use, we compared the SHE scale with TTO scenarios. In the following analyses, patients' data from all language centres (German, French and Italian speaking) were included. The comparison of SHE scores from the German and other centres showed that the scoring behaviour in all three language areas was sufficiently similar to justify merging the data.

Table 2 shows mean SHE scores for each possible value of TTO and for grouped values of TTO (12 vs 9 or 11 vs 3 or 6 vs 0 or 1 month) corresponding to 'perfect, good, fair and poor' utilities. The test for trend was highly significant in both analyses ($P < 0.001$).

### Influence of patient characteristics and treatment on SHE

SHE scores were significantly different in patient groups according to time since diagnosis (see Table 1). The mean SHE scores (s.d.) were 61.7 (20.0) for 11 patients with diagnosis in the previous 2 years, 52.3 (28.8) with diagnosis between 2 and 5 years (36 patients), 54 (23.5) with diagnosis between 5 and 10 years (26 patients) and 78.9 (18.5) with diagnosis more than 10 years before interview (11 patients) (ANOVA $P = 0.02$). Age reached borderline significance with older patients (> 56 years, 38 patients), showing lower mean values (53 vs 61) on the SHE scale (ANOVA $P = 0.04$). Treatment type also affected SHE scores. Mean score for 36

© Cancer Research Campaign 1998

patients receiving hormonal or no treatment was 62.8 (23.4), while it was 65.1 (22.1) for 15 patients receiving mild chemotherapy and 48.3 (28.5) for 33 patients receiving intensive chemotherapy (ANOVA $P = 0.03$).

### Comparison of SHE with HRQL LASA scores

In the total sample, in order to assess discriminant validity of SHE (i.e. whether this scale assesses something substantially different than the other LASA scales), correlations and tests for trend were also performed for all HRQL scales (physical well-being, appetite, mood and coping) with TTO to compare the strength of their associations. There was no significant association/trend between the TTO and the LASA scales for mood and coping. Although the association between SHE and TTO was stronger (linear-trend test $P \leq 0.001$), the scales for physical well-being and appetite also showed a significant linear-trend association with TTO ($P = 0.03$ and 0.01 respectively). Patients seemed to be more willing to trade time in current health when their physical condition was less good. The correlation between physical well-being or appetite and SHE was moderate ($r = 0.51$ and 0.42 respectively).

### Transformation of SHE-TTO

In a final step, we explored a power transformation of SHE scores to TTO utility scores (SHE-TTO) and vice versa (TTO-SHE). In the 48 German-speaking patients, and in contrast to others (Stiggelbout et al, 1996), we obtained a satisfactory fit at the individual patient level ($r^2 = 0.65$) for the SHE-TTO. Our estimate of alpha was 0.42 (s.d. 0.05), similar to a previous estimate of 0.47 (van Busschbach, 1994) for the same type of scale. Treating TTO as the dependent variable (TTO-SHE), we obtained an alpha of 1.54 (s.d. 0.17).

### DISCUSSION

Assigning values to individual health states is important to the assessment of many medical interventions. Both TTO and rating scales have been used in clinical studies. Coates and Simes (1992), Ashby et al (1994) and McQuellon et al (1995) studied patients with breast cancer. Stiggelbout et al (1994) and Boyd et al (1990) have studied disease-free testicular and gastrointestinal cancer patients. Feeny et al (1992) interviewed survivors of childhood cancer. Consecutive cancer patients were studied by Kiebert et al (1993) and Tsevat et al (1990). One of the main problems in obtaining evaluations of particular health states from persons not afflicted by the condition is finding a reliable and valid description of the health states (Llewellyn et al, 1982; Gerard et al, 1993; Ashby et al, 1994). Our purpose was limited to describing each patients' current health status, so this difficulty did not arise.

TTO and standard-gamble interviews are not feasible in the setting of large clinical trials, hence self-administered scales are an attractive alternative, if they work. In the present study, we have provided good evidence that the SHE scale is a valid and feasible method to assess the value patients attribute to their current state of health. As we expected, the SHE scale, anchored with worst health, was more acceptable to patients than SHED, although the death anchor point on SHED is more closely similar to classical utility. In fact, the two scales yielded closely similar values, indicating construct validity for SHE (Mummendey, 1987, pp. 104–109). Criterion validity is supported if the individual patient's answer on the scale corresponds with relevant outside criteria, assessed independently of the values given on the health estimation scale (Mummendey, 1987, pp. 81–83). We have shown criterion validity by demonstrating plausibly different mean values of SHE for groups of patients with different treatments and age. Different times since diagnosis as an indication of aggressiveness of disease also had a substantial impact on SHE scores. However, the trend of this association was not linear and needs further study. Our results showed that the answers that patients gave on both scales (SHE and SHED) were comparable.

To determine whether SHE measures something different to the other LASA scales used in the HRQL questionnaire, we compared all LASA scales with TTO answers. As the relationship between SHE and TTO was stronger than between the other LASA scales and TTO, this would give an indication of discriminant validity of SHE (Mummendey, 1987, pp. 84–85; Streaner and Norman, 1995).

The more time in their current health state that patients were prepared to trade for a lesser amount of time in perfect health, the lower were their health-state-estimation values on the SHE scale. This linear trend was highly significant. Our estimate of alpha for the SHE-TTO transformation (0.42, s.d. 0.05) was similar to that obtained by van Busschbach (1994), who also used an anchor of 'worst imaginable health' rather than 'death', but lower than the value proposed by Torrance (1986) and by Stiggelbout et al (1996); these two studies used a scale anchored by death and perfect health. Thus, the appropriate value of alpha for transformation may depend on the type of scale used, but both types of scale seem to allow transformation from LASA to TTO scores, so that patient self-assessment scores can be transformed for use in decision models. Additional research is needed to explain the observed power relationship (including the validity of the transformation). Different disease settings, being on treatment or not and other factors could be influencing this power function. Before using such a transformation, sensitivity analyses should be carried out and careful evaluation of small changes in the coefficient on the TTO should be performed.

While SHE had the most consistent relationship with TTO, a significant association was also found between the physical aspects of HRQL and the answers to the TTO questions. Patients seemed to be more prepared to trade some time in their current state of health when their physical condition was less good.

An open issue with regard to SHE is its subjective meaning to the individual patient filling in the scale. This issue is relevant generally in HRQL assessment and has been addressed by several authors (Schag et al, 1984; Slevin et al, 1988; Jenkins, 1992). We attempted to shed some light on this by asking patients about their thoughts when completing the scale. Their answers indicated some heterogeneity, as suspected by Jancik and Yates (1986). However, a large proportion of our patients scored the scale on the basis of thoughts about their current health.

Denial is a common coping style among cancer patients; this may affect SHE scores, as could be seen from the much higher means on SHE for the unrealistically optimistic patients and the divergence between SHE and SHED for patients who reacted unfavourably to the concept of death on the SHED scale. This may also apply to other self-assessment HRQL scales.

Our patients with metastatic breast cancer assessed their health state as relatively high. More than half of the patients would have traded no or only a very short time of their current condition for

© Cancer Research Campaign 1998

time in perfect health. This strong weighting of survival time per se is also reflected by the tendency to readily accept very discomforting health states with small gains in survival time observed in other studies with breast cancer patients (Coates and Simes, 1992; McQuellon et al, 1995).

We conclude from our study of the SHE scale in a limited sample of patients with metastatic breast cancer from three cultures that this simple scale is a feasible and valid method to assess the value patients attribute to their current state of health, especially for large-scale cancer clinical trials including patients from various countries and cultures. We have provided strong evidence that it can be transformed to give an adapted concept of utility, which can be incorporated as an approximation into quality-adjusted survival analysis, such as QALYs (Miyamoto, 1985; Carr-Hill, 1989; Mehrez, 1989) or Q-TWiST (Gelber and Goldhirsch, 1986; Goldhirsch et al, 1989). As SHE is patient derived, it is arguably preferable to arbitrary utility values assigned by investigators. We are currently assessing subjective health estimations by SHE in the large-scale international adjuvant breast cancer trials of the International Breast Cancer Study Group, in parallel with our conventional HRQL indicators. These studies will give us the opportunity to further investigate this scale, with special emphasis on cross-cultural factors, sensitivity to changes over time from diagnosis and the impact of different treatments and of disease relapse.

### ACKNOWLEDGEMENTS

We would like to thank the physicians, nurses and patients who participated in the study and L Bacchus, M Tomamichel and Mrs Warrick who interviewed patients in St Gallen, Lugano and Geneva. We are grateful to M Castiglione, Scientific Director of the SAKK/SIAK, for providing the infrastructure of the Coordinating Centre. We extend our special thanks to S Pampallona, G Jones, R Gelber and A Goldhirsch for reviewing and commenting on earlier versions of this paper.

### REFERENCES

Altman DG and Bland JM (1983) Measurement in medicine: the analysis of method comparison studies. *Statistician* **32**: 307–317

Ashby J, O'Hanlon M and Buxton MJ (1994) The time trade-off technique: how do the valuations of breast cancer patients compare to those of other groups? *Qual Life Res* **3**: 257–265

Boyd NF, Sutherland H, Heasman K, Tritchler D and Cummings B (1990) Whose utilities for decision analysis? *Med Decis Making* **10**: 58–67

Bradley EL (1985) Overlapping coefficient. In *Encyclopedia of Statistical Sciences* Vol. 6, Kotz S and Johnson NL. (eds), pp. 546–547, Wiley: New York

Carr-Hill R (1989) Background material for the workshop on QUALYs. Assumptions of the QUALY procedure. *J Sci Med* **29**: 469–477

Coates A and Simes RJ (1992) Patient assessment of adjuvant treatment in operable breast cancer. In *Introducing New Treatments for Cancer: Practical, Ethical and Legal Problems*. Williams CJ. (ed.), pp. 447–458. John Wiley & Sons: New York

Cole BF, Gelber R and Goldhirsch A (1995) Comparing treatments using quality-adjusted survival: the Q-TWiST method. *Am Statistician* **49**: 161–169

Cuzick J (1985) A Wilcoxon-type test for trend. *Stat Med* **4**: 87–90

Feeny DH and Torrance G (1989) Incorporating utility-based quality-of-life assessment measures in clinical trials. Two examples. *Med Care* **27** (suppl.): S190–S204

Feeny DH and Furlong W, Barr R, Torrance GW, Rosenbaum P and Weitzman S (1992) A comprehensive multiattribute system for classifying the health status of survivors of childhood cancer. *J Clin Oncol* **10**: 923–928

Gelber RD and Goldhirsch A (1986) A new endpoint for the assessment of adjuvant therapy in postmenopausal women with operable breast cancer. *J Clin Oncol* **4**: 1772–1779

Gelber RD, Cole BF, Goldhirsch A, Rose C, Fisher B, Osborne CK, Boccardo F, Gray R, Gordon NH, Bengtsson NO and Sevelda P (1996) Adjuvant chemotherapy plus tamoxifen compared with tamoxifen alone for postmenopausal breast cancer: meta-analysis of quality-adjusted survival. *Lancet* **347**: 1066–1071

Gerard K, Dobson M and Hall J (1993) Framing and labelling effects in health descriptions: quality adjusted life years for treatment of breast cancer. *J Clin Epidemiol* **46**: 77–84

Goldhirsch A, Gelber R, Simes J, Glasziou P and Coates A for the Ludwig Breast Cancer Study Group (1989) Costs and benefits of adjuvant therapy in breast cancer: a quality-adjusted survival analysis. *J Clin Oncol* **7**: 36–44

Guyatt GH, Feeny DH and Patrick DL (1993) Measuring health-related quality of life. *Ann Intern Med* **118**: 622–629

Hürny C, Bernhard J, Gelber R, Coates A, Castiglione M, Isley M, Dreher D, Peterson H, Goldhirsch A and Senn HJ for the International Breast Cancer Study Group (1992) Quality of life measures for patients receiving adjuvant therapy for breast cancer: an international trial. *Eur J Cancer* **28**: 118–124

Hürny C, Bernhard J, Bacchi M, Van Wegberg B, Tomamichel M, Speck U, Coates A, Castiglione M, Goldhirsch A and Senn HJ for the Swiss Group for Cancer Research and the International Breast Cancer Study Group (1993) The perceived adjustment to chronic illness scale (PACIS): a global indicator of coping for operable breast cancer patients in clinical trials. *Supp Care Cancer* **1**: 200–208

Hürny C, Bernhard J, Coates AS, Castiglione-Gertsch M, Peterson HF, Gelber RD, Forbes JF, Rudenstam CM, Simoncini E, Crivellari D, Goldhirsch A and Senn HJ for the International Breast Cancer Study Group (1996) Impact of adjuvant therapy on quality of life in women with node-positive operable breast cancer. *Lancet* **347**: 1279–1284

Inman HF and Bradley EL Jr (1989) The overlapping coefficient as a measure of agreement between 2 probability distributions and point estimation of the overlap of two normal densities. *Commun Stat Theory Methodol* **18**: 3851–3874

Jancik R and Yates JW (1986) Quality of life assessment of cancer patients: conceptual and methodological challenges and constraints. *Cancer Bull* **38**: 217–222

Jenkins D (1992) Assessment of outcomes of health intervention. *Soc Sci Med* **35**: 367–375

Kaplan RM and Anderson JP (1990) The general health policy model: an integrated approach. In *Quality of Life Assessments in Clinical Trials* Spilker B. (ed.), pp. 131–149. Raven Press: New York

Kiebert G, Stiggelbout AM, Leer JW, Kievit J and De Haes HJ (1993) Test-retest reliabilities of two treatment preference instruments in measuring utilities. *Med Decis Making* **13**: 133–140

Llewellyn-Thomas H, Sutherland HJ, Tibshirani R, Ciampi A, Till JE and Boyd NF (1982) The measurement of patients' values in medicine. *Med Decis Making* **2**: 449–462

McQuellon RP, Hyman BM, Hoffman SL, Russel G, Craven B and Yellen S (1995) Patient preferences for treatment of metastatic breast cancer: a study of women with early-stage breast cancer. *J Clin Oncol* **13**: 858–568

Mehrez A and Gafni A (1989) Quality-adjusted life years, utility theory and healthy-years equivalents. *Med Decis Making* **9**: 142–149

Miyamoto JM and Eraker StA (1985) Parameter Estimates for a QUALY utility model. *Med Decis Making* **5**: 191–213

Mummendey HD (1987) *Die Fragebogen-Methode*. Hogrefe: Göttingen

Noack H (1990) Gemeinsame Betrachtung von Lebenszeit und Lebensqualität, Poster auf der Arbeitstagung 'Erfassung von Lebensqualität in der Onkologie', Heidelberg, 10–12 Mai 1990

Read JL, Quinn RL, Berwick DM, Fineberg HB and Weinstein M (1984) Preferences for health outcomes. Comparison of assessment methods. *Med Decis Making* **4**: 315–329

Ross R and Kind P (1978) A scale of valuations of states of illness: is there a social consensus? *Int J Epidemiol* **7**: 347–358

Schag CC, Heinrich RL and Ganz PA (1984) Karnofsky performance status revisited: reliability, validity and guidelines. *J Clin Oncol* **2**: 187–193

Slevin ML, Plant H, Lynch D, Drinkwater J and Gregory WM (1988) Who should measure quality of life, the doctor or the patient? *Br J Cancer* **57**: 109–112

Snedecor GW and Cochran WG (1989) *Statistical Methods*. 8th edn. Iowa State University Press: Ames, IA

Stiggelbout AM, Kiebert GM, Kievit J, Leer JWH, Stoter G and De Haes JC (1994) Utility assessment in cancer patients: adjustment of time tradeoff scores for the utility of life years and comparison with standard gamble scores. *Med Decis Making* **14**: 82–90

Stiggelbout AM, Eijkemans MJ, Kiebert GM, Kievit J, Leer, JW and De Haes HJ (1996) The 'utility' of the visual analog scale in medical decision making and

 © Cancer Research Campaign 1998

technology assessment. Is it an alternative to the time trade- off? *Int J Technol Assess Health Care* **12**: 291–298
Streaner and Norman (1995) *Health Measurement Scales. A Practical Guide to their Development and Use*. 2nd edn. University Press: Oxford, UK
Sutherland H, Dunn V and Boyd N (1983) Measurement of values for states of health with linear analog scales. *Med Decis Making* **3**: 477–487
Torrance G (1976) Social preferences for health states: an empirical evaluation of three measurement techniques. *Socio-Economic Planning Sciences* **10**: 129–136
Torrance G (1986) Measurement of health state utilities for economic appraisal. A review. *J Health Econ* **5**: 1–30
Torrance G (1987) Utility approach to measuring health-related quality of life. *J Chron Dis* **40**: 593–600
Tsevat J, Dawson N and Matchar D (1990) Assessing quality of life and preferences in the seriously ill using utility theory. *J Clin Epidemiol* **43** (suppl.): 73S–77S.
Van Busschbach J (1994) De validiteit van QUALY's (The validity of QUALY's). PhD thesis. Erasmus University Rotterdam, Sanders Instituut: Rotterdam
Weeks J (1991) Utilities as a measure of quality of life in cancer patients. Study documentation for an investigation conducted for the Dana-Farber Cancer Institute, Boston

### APPENDIX 1

### Utility and quality of life LASA scales

Patient's name: ____________Date: _____Center:______Int. __

Please spare a moment to answer the following questions. Your information will be treated as strictly confidential. Thank you for replying.

Please place a vertical line according to how you rate the following aspects overall, for the entire period since your last full clinical assessment.

### Example:

*Tiredness:*
None ____________________________________________A lot

*Physical well-being:*
Good ___________________________________________Lousy

*Mood:*
Happy ________________________________________Miserable

Imagine you had to spend the rest of your life in your current condition. How would you rate such a life between:
Perfect health ___________________________________Death

*Appetite:*
Good _____________________________________________None

*How much effort does it cost you to cope with your illness:*
No effort at all ______________________________A great deal

Imagine you had to spend the rest of your life in your current condition. How would you rate such a life between:

Perfect health ______________________Worst possible health

### APPENDIX 2

### Time trade-off interview

Patient's name: ____________Date: _____Center:______Int. __

(Instructions to Interviewer are always in brackets.)
1. Comprarison of health state today with varying amounts of survival time in perfect health (Interview developed by Jane Weeks, Dana-Farber Cancer Institute, Boston).

Introduction: I am now going to ask you a few questions dealing with how you feel about your current state of health. Please realize that these questions do not mean that anyone knows how long you will live, we simply want to know what you would do if you had a choice.

For example, if I ask you whether you would prefer spending one year in your current state of health or one year in excellent health, you would probably answer one year in excellent health.

(Interviewer: 'indifferent' or 'don't care' means that the respondent cannot decide because he/she sees the two options as equal, not that he/she does not understand or refuses to answer the question).

Now please tell me which you would prefer:

Living 1 year in your current state of health, living 11 months in excellent health, or do you consider the two choices equal?

— 1 year in current health
— 11 months in excellent health
— Equal (indifferent, no preference)

How about:

— 1 year in current health
— 9 months in excellent health
— Equal (indifferent, no preference)

How about:

— 1 year in current health
— 6 months in excellent health
— Equal (indifferent, no preference)

How about:

— 1 year in current health
— 3 months in excellent health
— Equal (indifferent, no preference)

How about:

— 1 year in current health
— 1 month in excellent health
— Equal (indifferent, no preference)

© Cancer Research Campaign 1998